# Medical Students’ Perceptions and Attitudes Toward English as a Medium of Instruction at the Faculty of Medicine and Pharmacy of Rabat: Cross-Sectional Study

**DOI:** 10.2196/95392

**Published:** 2026-07-23

**Authors:** Ilham Maaroufi, Rachid Razine, Zhor Zeghari, Ikbal Zeddari, Driss Laayadi, Majdouline Obtel, Jihane Belayachi, Redouane Abouqal

**Affiliations:** 1 Laboratory of Biostatistics, Clinical Research, and Epidemiology, Faculty of Medicine and Pharmacy, Mohammed V University in Rabat, Rabat, Morocco; 2 Department of Cardiovascular Surgery, Ibn Sina University Hospital, Impasse Souissi, Rabat, 10100, Morocco, 212 5 37 77 29 81; 3 Laboratory of Community Health (Public Health, Preventive Medicine, and Hygiene), Department of Public Health, Faculty of Medicine and Pharmacy, Mohammed V University in Rabat, Rabat, Morocco; 4 Department of English, Faculty of Letters and Human Sciences, Mohammed V University in Rabat, Rabat, Morocco; 5 Acute Medical Unit, Ibn Sina University Hospital, Rabat, Morocco

**Keywords:** English-medium instruction, medical education, students’ perceptions, attitudes, Morocco, Global South

## Abstract

**Background:**

The global adoption of English-medium instruction (EMI) in higher education has introduced complex implementation challenges, the severity of which often depends on the resources available within specific educational contexts. Evidence remains limited in public medical schools in under-resourced, non-Anglophone countries, which serve a socioeconomically and educationally diverse student population. In such settings, the direct transfer of existing EMI integration models presents significant practical challenges.

**Objective:**

This exploratory, observational, monocentric study aimed to investigate medical students’ perceptions and attitudes toward EMI implementation at the Faculty of Medicine and Pharmacy of Rabat (FMPR) in Morocco and to evaluate their readiness for EMI adoption.

**Methods:**

A cross-sectional survey was administered to 102 second-cycle medical students at FMPR. The 23-item questionnaire included Likert-type scales and multiple-choice questions across 4 domains: demographic data, self-reported English language proficiency, language perceptions and attitudes, and EMI needs. Bivariate, univariate, and multivariate logistic regression analyses were conducted to determine the effects of the explanatory variables on EMI choice.

**Results:**

The sample comprised 65 female and 37 male participants. Most reported using English speaking and listening skills “often” on a daily basis, while writing skills were reported as being used “rarely.” Participants rated their general English proficiency and their academic listening and reading skills as “good.” Overall, 82.4% (84/102) of students were strongly in favor of implementing EMI at FMPR. Bivariate analysis showed significant positive associations between EMI choice and age (*P*=.04), course year (*P*<.001), interest in learning English (*P*=.006), and the belief that English should be the language of higher education (*P*=.02). Preference for EMI increased alongside course year, peaking at 96.4% (27/28) among students in the third and fourth years. Multivariate logistic regression confirmed that being in the fourth vs third course year (adjusted odds ratio 22.12, 95% CI 2.56‐190.84; *P*=.005) and having an interest in learning English (adjusted odds ratio 5.12, 95% CI 1.05‐25.03; *P*=.04) were significant positive predictors of EMI choice.

**Conclusions:**

Medical students at FMPR exhibit highly positive perceptions and attitudes toward the potential implementation of EMI, despite variations in their self-reported English readiness. These findings provide actionable insights for the successful integration of EMI that extend beyond the Moroccan context, offering a valuable framework for medical institutions in other under-resourced, low- and middle-income, non-Anglophone settings across the Global South.

## Introduction

### Conceptual Framework: Epistemic Justice and Constructivism

The 21st century has witnessed the consolidation of English as the global lingua franca for science, technology, and medicine, driving the EMI expansion across higher education systems in Europe, Asia, and beyond [1-7]. However, while the adoption of English-medium instruction (EMI) is rapidly expanding globally, public medical schools in low-income and middle-income non-Anglophone countries often face significant structural and pedagogical barriers to its equitable implementation [4,7-9].

Therefore, to critically analyze the EMI adoption in postcolonial, resource-constrained settings, it is essential to establish a conceptual foundation at the outset. As R’boul [10] cautions, language policies in the Global South are deeply intertwined with linguistic dependency and epistemic justice [11,12]. The uncritical imposition of a foreign language can be prejudicial to vulnerable students [13]. Consequently, this study adopts a constructivist understanding, which posits that Moroccan students are not passive recipients of top-down educational directives [14-17]. Instead, their perceptions and attitudes toward EMI implementation are actively shaped by their sociolinguistic environment, balancing historical linguistic inequities with the democratizing promise of English.

### Global Expansion of EMI

Viewed from this perspective, a review of the empirical literature regarding students’ perceptions of EMI reveals a complex global landscape. In higher education, EMI has been increasingly adopted in non-Anglophone settings to enhance students’ access to global communication, employability, and institutional competitiveness [2-5]. In Europe, medical schools are increasingly adopting EMI to standardize curricula and drive the internationalization of medical education [18,19]. In Asia, universities leverage EMI to foster intercultural exchange and bolster their international reputation within an expanding competitive market [3,20,21]. Across Africa, EMI adoption in higher education extends beyond former British colonies, with countries previously colonized by France, Germany, and Portugal embracing English as a gateway to globalized higher education [22,23].

### EMI in the Middle East and North Africa Region

This trend is expanding into the Middle East and North Africa region, where the introduction of EMI in medical education is increasingly viewed as a necessary bridge to align health care systems with global scientific standards and facilitate the acquisition of indispensable medical terminology [14,24]. Middle-Eastern countries are adopting EMI in medical schools as part of broader strategic transitions toward knowledge-based economies. In their medical schools, EMI is dominant and often supported by favorable student attitudes, as observed in Saudi Arabia [25-27]. While students report advantages, they also often face challenges when transitioning from Arabic-language high school education to English-medium higher education [7,25,28].

Although English is well-established in Egyptian medical education due to British historical influence, countries like Libya are cautiously adopting EMI, even though Arabic remains the dominant instructional medium [9,29,30]. In Francophone North-African countries, despite French retaining its dominance in higher education due to linguistic policies inherited from French colonialism, EMI programs are progressively embraced in medical schools to meet international medical standards [11,15,31,32].

### The Moroccan Context: Sociolinguistic Duality and Student Attitudes

In Morocco, EMI implementation creates a sociolinguistic duality that mirrors broader patterns observed across North African higher education systems, where English is progressively being promoted [22,29]. Research indicates a strong general enthusiasm for English among Moroccan students and highly positive perceptions toward EMI [16,17,33-36]. However, recent empirical studies in Moroccan higher education have reported that many students frequently express anxiety regarding their English proficiency, preparedness, and the adequacy of institutional support [16,17].

To understand these ambivalent attitudes, where enthusiasm for English coexists with academic anxiety, it is essential to examine the unique paradox of the Moroccan sociolinguistic landscape. Historically, the enduring dominance of French as the sole language of science has acted as a barrier to educational equity. This dominance favors students from privileged socioeconomic backgrounds who have access to costly French-medium private primary and secondary schooling, which significantly impacts academic performance [13,15,37]. Conversely, English is largely devoid of colonial connotations for Moroccan youth and is increasingly democratized through digital engagement and globalized media, fostering basic interpersonal communication skills among a highly favorable youth population [15,33,36].

Aligning with this evolving sociocultural reality and recent United Kingdom–Morocco educational agreements aimed at enhancing English integration, Morocco is strategically advancing the status of English within its educational system through the 2022‐2026 Educational Roadmap [33,38-40]. The primary goal of this reform is to enhance systemic competitiveness by strengthening English proficiency across higher education [33,38]. Although EMI has the potential to foster epistemic justice and offer a more inclusive linguistic alternative for future physicians [7,10,15], its implementation in public medical education must be managed cautiously to mitigate dual cognitive burdens and ensure adequate preparedness of both educators and students [8,10,37].

Furthermore, at the individual level, sociodemographic factors, specifically sex, are theoretically relevant when analyzing students’ attitudes. Recent meta-analyses indicate that while sex is a frequently examined variable, sex-related differences in language learning anxiety are complex and not consistently statistically significant across all contexts [41]. Therefore, including sex in our statistical analysis allows us to empirically test its specific influence within the Moroccan medical EMI context.

### Research Gap and Study Objectives

While several universities are diversifying their pedagogical offerings to attract international students, research on EMI readiness in Moroccan public medical schools like the Faculty of Medicine and Pharmacy of Rabat (FMPR) remains notably scarce [32,34,36]. To the best of our knowledge, no formative studies have evaluated students’ perceptions and attitudes toward EMI implementation specifically within a public medical school like FMPR. Unlike privileged private institutions, FMPR attracts a socioeconomically diverse population, many of whom lack the financial resources required for supplemental academic English training. This presents a critical research gap with profound implications for equitable access to globalized medical education, positioning FMPR as a critical case study to explore EMI implementation challenges in public medical education.

The urgency of EMI integration in the Global South is amplified by international accreditation pressures. The World Federation for Medical Education (WFME) now underpins the global recognition of medical school accreditation [42]. Starting in 2024, the Educational Commission for Foreign Medical Graduates requires that applicants to the United States Medical Licensing Examination graduate from a medical school accredited by a WFME-recognized agency [43]. Public faculties, which are lagging behind private institutions in EMI adoption, risk severely limiting their students’ global mobility and career prospects.

To address these systemic needs and explore the potential for equitable EMI integration, this formative study investigates medical students’ perceptions and attitudes toward EMI implementation at FMPR, alongside their self-perceived English readiness. Using an exploratory, observational, and monocentric design, this research aims to answer the following questions:

1. What are the students’ perceived English proficiency across the 4 language skills, particularly in academic English?
2. What are the students’ perceptions and attitudes toward the potential integration of EMI at FMPR?
3. What factors underpin students’ EMI choices (sociodemographic and English-related attitudinal factors)?

By focusing on a socioeconomically diverse student population, this study provides actionable insights to guide evidence-based language policies in comparable Global South contexts.

## Methods

### Study Design

This research is an exploratory, observational, and monocentric cross-sectional study conducted at FMPR during the 2023‐2024 academic year. The study adhered to the STROBE (Strengthening the Reporting of Observational Studies in Epidemiology) guidelines (Checklist 1). An exploratory design was chosen because there is no prior research on EMI implementation within a Moroccan public medical faculty. The observational aspect focused on collecting descriptive data from the sample through a quantitative approach, using a questionnaire with Likert-type scale items and multiple-choice questions.

### Participants and Setting

The target population consisted of medical students enrolled at FMPR during the 2023‐2024 academic year. A convenience sampling method was used, resulting in a sample of 102 undergraduate medical students from the second cycle of the medical curriculum (third, fourth, fifth, and sixth years of study). This sample size was considered appropriate for an exploratory study aimed at identifying preliminary trends. The inclusion criteria encompassed all enrolled students who were able to complete the questionnaire in French (the main instructional medium at FMPR) or, where applicable, in English, provided they were proficient in either French or English. The exclusion criteria were limited to students who declined to participate or who submitted incomplete questionnaires. All 102 participants completed the French version of the questionnaire.

Demographically, the final sample consisted of 65 (63.7%) female and 37 (36.3%) male participants, with a median age of 21 (IQR 21‐23; range 20‐25 y) years and a mean age of 21.8 (SD 1.43) years. Regarding socioeconomic and educational backgrounds, FMPR encompasses a highly diverse student population. This includes individuals from modest socioeconomic backgrounds who attended public high schools, as well as those from privileged social classes with prior access to private French-medium schooling.

### Ethical Considerations

According to the Ethics Committee for Biomedical Research at FMPR and Article 2 of Moroccan Law No. 28‐13 [44], noninterventional observational research falls strictly outside the scope of formal biomedical ethics review. Accordingly, no formal ethics approval or exemption was applied for this educational survey, and no protocol number is applicable. Nevertheless, the study was conducted in strict accordance with the ethical principles of the Declaration of Helsinki. All participants provided written informed consent prior to participating. No identifying or sensitive personal information was collected, and all responses were gathered anonymously.

### Study Instrument

Data were collected using a custom-developed questionnaire divided into 4 sections. The first section gathered demographic data (3 items: age, sex, and course year). The second section (8 items) assessed students’ perceived proficiency in English, including their background, usage in general and academic contexts, and exposure across the 4 main language skills. For instance, students were asked to rate their perceived English proficiency on a scale of 0 to 5 for tasks such as “Reading medicine textbooks in English” and “Communicating in English with my course instructors.” The third section (1 matrix question comprising 9 Likert-scale items) explored perceptions and attitudes toward EMI. Items regarding perceived benefits and disadvantages were developed based on the existing literature [1-3,7,9,10,18,29,37]. Representative items from this section asked students to rate their agreement from 1 (“strongly disagree”) to 5 (“strongly agree”) on statements such as “English should be the language of higher education” and “If an English program is offered in my faculty, I will definitely go for it.” The fourth section investigated students’ future EMI needs. The complete self-administered questionnaire, including all items on demographic characteristics, perceived English proficiency, language perceptions and attitudes, and EMI needs, is provided in Multimedia Appendix 1. This study reports exclusively on data collected from the first 3 sections.

The questionnaire’s validity and reliability were established through rigorous, complementary methods. First, content and face validity were established through pilot testing among 15 participants from the target group, who assessed the items for clarity, relevance, and comprehensibility. Expert evaluation was subsequently conducted by 6 specialists, including 3 professors of medicine and methodology and 3 experts in English studies and EMI. The questionnaire was iteratively refined based on their recommendations until final approval was reached.

The questionnaire was developed as a shared instrument across 2 related studies examining complementary aspects of EMI in medical education. To assess the construct validity and dimensional structure of the perceived English proficiency items, we conducted an exploratory factor analysis (EFA) on the 17 items from Section II (Q8: general proficiency; Q9: academic proficiency; Q10: exposure to English) using maximum likelihood extraction with varimax rotation. The Language Perceptions and Attitudes scale (Section III, Q12, 9 items) was validated through expert content review and pilot testing and is reported descriptively as a separate content-validated scale.

Although the EFA confirmed the construct validity and dimensional structure of the perceived English proficiency items, we chose to present the descriptive results according to the original theoretical dimensions of the questionnaire, namely, Perceived English Proficiency and Language Perceptions and Attitudes toward EMI. We believe this approach better preserves the conceptual integrity of our framework and allows for more meaningful comparisons with previous EMI studies conducted in similar contexts.

### Data Collection

Data collection was conducted during the spring semester of the 2023‐2024 academic year (from March to May). The survey used self-administered, paper-based questionnaires, which were available in both French and English, allowing respondents to choose their preferred language version to maximize comprehension and response validity. Students received clear written instructions on how to appropriately fill out the Likert scales and multiple-choice items. A total of 130 questionnaires were distributed in person to medical students, primarily at the end of their lectures. They completed the survey in 10 to 15 minutes. Of these, 28 were incompletely answered and thus excluded, leaving 102 valid responses for the final analysis.

### Statistical Analysis

Statistical analysis was performed using the open-source software Jamovi (version 2.6.26; The Jamovi Project) [45]. For analytical purposes, all response scales were recoded into 3 categories prior to analysis. Items were then aggregated into dimension mean scores to measure the intended constructs: perceived proficiency in English, as well as language perceptions and attitudes toward EMI. To ensure data integrity, any questionnaire containing missing or incomplete responses was entirely excluded from the dataset prior to statistical analysis.

Descriptive statistics were calculated for all variables. The normality of quantitative variables was assessed using the Shapiro-Wilk test. Quantitative variables with non-Gaussian distributions were reported as medians and IQR, whereas qualitative variables were expressed as frequencies and percentages.

Bivariate analyses were conducted using the chi-square test or Fisher exact test, as appropriate. Univariate and multivariate logistic regression models were used to determine the effect of the explanatory variables on the dependent variable “EMI choice.” For the multivariate analysis, independent variables were initially selected based on their statistical significance in the preliminary bivariate analysis. Additionally, specific variables, such as sex and “English is useful for the medical field,” were forced into the initial model based on their contextual and theoretical relevance. A backward stepwise regression method was subsequently applied to retain the most robust statistical predictors. A *P* value <.05 was considered statistically significant. Associations were expressed as odds ratios (ORs) and adjusted odds ratios (aORs) with 95% CIs.

## Results

### EFA and Reliability

The data demonstrated excellent suitability for factor analysis (Kaiser-Meyer-Olkin [KMO]=0.903; Bartlett *χ*²_136_=1505; *P*<.001), yielding 2 factors that together explained 62.5% of the total variance. The internal consistency reliability of each dimension was subsequently assessed using Cronbach α, with a cut-off of 0.50 considered acceptable for exploratory research [46,47] (Table 1).

**Table 1.** Psychometric properties of the exploratory factor analysis (EFA) factors and the questionnaire dimensions^a^.

Psychometric properties	Items, n	Factor loadings	Cronbach α
EFA derived factors			
F1: Academic English proficiency (question 9)	7	0.660-0.905	0.943
F2: General English proficiency and exposure (questions 8 and 10)^b^	10	0.566-0.811	0.920
Questionnaire dimensions			
Perceived English proficiency (F1+F2)	17	—^c^	0.949
Language perceptions and attitudes toward EMI^d^^,^^e^	7	—	0.670

a Kaiser-Meyer-Olkin [KMO]=0.903; Bartlett *χ*²_136_=1505; *P*<.001; total variance explained=62.5%.

b Item “Private English classes” was excluded from factor 2 due to high uniqueness (0.898), indicating insufficient shared variance with the other exposure items.

c Not applicable.

d “Language Perceptions and Attitudes toward EMI” is reported descriptively as a content-validated scale, not included in the EFA. The item “I like learning English” was excluded due to conceptual redundancy with the item “I like to use English.” The item “We should keep both French and English as mediums of instruction” was removed following reliability analysis, as it correlated negatively with the total scale (α without item=0.670 vs α with item=0.483), suggesting it measures a conceptually distinct construct.

e EMI: English-medium instruction.

### Participant Characteristics

Regarding academic progression, among 102 students, 39 (38.2%) were in their third year of study, 28 (27.5%) in their fourth year, 20 (19.6%) in their fifth year, and 15 (14.7%) in their sixth year. The vast majority of participants (n=95, 93.1%) reported speaking English in their daily lives. Over half (n=53, 52.0%) started learning English in primary school, while 35 (34.3%) began in secondary school. However, only 29 (28.4%) students held an English certification aligned with the Common European Framework of Reference for Languages (CEFR), specifically at B2 (n=10, 9.8%), C1 (n=10, 9.8%), and C2 (n=5, 4.9%) levels.

### Students’ Perceived English Proficiency

#### Exposure to English

Participants reported “moderate to frequent exposure” to English (7 items; mean score 2.28/3, range reporting “often” 12.7%‐87.3%; Table 2). All participants declared frequently (“often”) practicing speaking and listening skills through habits such as interacting with friends, watching television, and listening to English music or podcasts. Conversely, exposure to writing in English was reported as “rare” (Table 2).

**Table 2.** Students with exposure to English (N=102).

Activity	Rarely, n (%)	Sometimes, n (%)	Often, n (%)
Interacting with friends	18 (17.6)	32 (31.4)	52 (51)
Watching movies	5 (4.9)	8 (7.8)	89 (87.3)
Listening to the radio/music	6 (5.9)	8 (7.8)	88 (86.3)
Listening to podcasts	10 (9.8)	25 (24.5)	67 (65.7)
Reading	13 (12.7)	40 (39.2)	49 (48.0)
Free writing	53 (52)	32 (31.4)	17 (16.7)
Private English classes	69 (67.6)	20 (19.6)	13 (12.7)

#### General and Academic Perceived English Proficiency

Regarding general English competencies, self-reported proficiency was largely positive (4 items; mean score 2.58/3, range rating “good” 60.8%‐75.5%). The majority of participants evaluated their abilities as “good” across routine tasks, including listening, reading, writing on social media, and speaking during everyday interactions.

In contrast, perceptions of academic English proficiency were notably lower (7 items; mean score 2.05/3, range rating “average” to “good” 19.6%‐53.9%). Participants rated their receptive skills (listening and reading) as “good,” whereas their productive skills (speaking and writing) were considered “average.” Specifically, the majority of students considered their ability to write a scientific research study as “basic.” A minority of respondents expressed apprehension regarding specific academic tasks, rating their skills as “basic” for understanding videos on medical issues (11/102, 10.8%), comprehending English-medium medical textbooks (17/102, 16.7%), writing academic assignments in English (25/102, 24.5%), participating in class discussions (29/102, 28.4%), and preparing and delivering presentations (31/102, 30.4%; Table 3).

**Table 3.** Perceived students’ skills in general and academic English (N=102).

Skills	Basic, n (%)	Average, n (%)	Good, n (%)
General English			
Speak in regular everyday interactions	10 (9.8)	30 (29.4)	62 (60.8)
Listen in regular daily interactions	7 (6.9)	18 (17.6)	77 (75.5)
Read about general topics of interest to me (newspapers, magazines, and books)	9 (8.8)	26 (25.5)	67 (65.7)
Communicate in writing on social media platforms	7 (6.9)	26 (25.5)	69 (67.6)
Academic English			
Discuss technical ideas in the field of medicine	29 (28.4)	46 (45.1)	27 (26.5)
Prepare and deliver a presentation about a medical issue	31 (30.4)	47 (46.1)	24 (23.5)
Understand videos about medical issues	11 (10.8)	36 (35.3)	55 (53.9)
Read medicine textbooks written in English	17 (16.7)	36 (35.3)	49 (48.0)
Write class assignments in English about topics in medicine	25 (24.5)	50 (49)	27 (26.5)
Communicate in English with my course instructors	30 (29.4)	45 (44.1)	27 (26.5)
Write a scientific article for publication in a medical journal	44 (43.1)	38 (37.3)	20 (19.6)

### Students’ Perceptions and Attitudes Toward English and EMI

#### Perceptions and Attitudes Toward English and EMI

Participants demonstrated favorable perceptions and attitudes toward English and EMI implementation in medical education (9 items; mean score 2.83/3, range of “agree” 64.7%‐98.0%; Table 4). Among 102 students, 98 (96.1%) agreed that English is useful in the medical field, and 90 (88.2%) believed that English should be the language of higher education. However, 74 (72.5%) students expressed a preference for maintaining a bilingual medical curriculum, using both French and English as media of instruction at the faculty.

**Table 4.** Students’ perceptions and attitudes (N=102).

Statement	Agree, n (%)	Neutral, n (%)	Disagree, n (%)
I like to learn English	91 (89.2)	7 (6.9)	4 (3.9)
I like to use English	85 (83.3)	15 (14.7)	2 (2.0)
English is very useful for work in Morocco	66 (64.7)	35 (34.3)	1 (1.0)
English is useful for the medical scientific field	98 (96.1)	4 (3.9)	—^a^
English should be the language of higher education	90 (88.2)	11 (10.8)	1 (1.0)
We should have more courses using EMI^b^ in higher education	89 (87.3)	13 (12.7)	—
If an EMI program is offered in my faculty, I will definitely go for it	84 (82.4)	18 (17.6)	—
Multilingualism offers me advantages	100 (98.0)	2 (2.0)	—
We should keep both French and English as mediums of instruction in higher education	74 (72.5)	25 (24.5)	3 (2.9)

a Not applicable.

b EMI: English-medium instruction.

#### Perceived Benefits and Disadvantages of EMI

Participants recognized significant advantages to EMI implementation (4 items; mean score 2.48/3, range of “yes” 65.7%‐83.2%; Table 5). Specifically, respondents indicated that EMI would facilitate international mobility (85/102, 83.3%), provide better professional opportunities (82/102, 80.4%), enhance the learning of medical content (71/102, 69.6%), and improve their English skills (67/102, 65.7%).

Conversely, perceived disadvantages received lower rates of agreement (4 items; mean score 1.30/3, range of “yes” 2.9%‐36.3%; Table 5). Reported concerns included the potential difficulty of learning medical subjects in English (37/102, 36.3%), the erosion of the native language (15/102, 14.7%), the loss of local culture (8/102, 7.8%), and the loss of personal identity (3/102, 2.9%).

**Table 5.** Perceived benefits and disadvantages of English-medium instruction (EMI) according to students (N=102).

Statement	Yes, n (%)	No, n (%)
Advantages		
To improve my level of English skills (listening, speaking, oral interactions, presentations, debates, reading, writing)	67 (65.7)	35 (34.3)
To improve my learning of the medical content subjects	71 (69.6)	31 (30.4)
To have better professional opportunities	82 (80.4)	20 (19.6)
To have more opportunities regarding international mobility (study or work abroad)	85 (83.2)	17 (16.8)
Disadvantages		
Loss of mother tongue^a^	15 (14.7)	87 (85.3)
Loss of local culture	8 (7.8)	94 (92.2)
Loss of identity	3 (2.9)	99 (97.1)
The content subjects are more difficult to learn in English	37(36.3)	65 (63.7)

a The original questionnaire item used the term “mother tongue,” which is referred to as “native language” in the text.

### Factors Influencing Students’ EMI Choice

A bivariate analysis was conducted to examine the association between the explanatory variables and the dependent variable representing EMI choice (“If an EMI course is offered in my faculty, I will definitely go for it”). Statistically significant associations were observed between EMI choice and age (*P*=.04), course year (*P*<.001), the variable “I like learning English” (*P*=.006), and the variable “English should be the language of higher education” (*P=*.02; Table 6). The proportion of EMI choice increased with academic progression, rising from 53.8% (21/39) in the third year to 96.4% (27/28) in the fourth year. High percentages of EMI preference were also observed among students who agreed with “I like learning English” (69/91, 75.8%) and “English should be the language of higher education” (68/90, 75.6%). Sex and English certification did not reach statistical significance.

**Table 6.** Bivariate analysis between English-medium instruction (EMI) choice and the explanatory variables (N=102).

Variable	EMI choice		*P* value
	Yes, n (%)	No, n (%)	
Age (y)			.04
18-20	14 (51.9)	13 (48.1)	
21-23	42 (75.0)	14 (25.5)	
24-26	15 (88.2)	2 (11.8)	
≥27	2 (100.0)	—^a^	
Sex			.25
Male	29 (78.4)	8 (21.6)	
Female	44 (67.7)	21 (32.3)	
Course year			<.001
Third	21 (53.8)	18 (46.2)	
Fourth	27 (96.4)	1 (3.6)	
Fifth	14 (70.0)	6 (30.0)	
Sixth	11 (73.3)	4 (26.7)	
English certification			.28
Certified	23 (79.3)	6 (20.7)	
Noncertified	50 (68.5)	23 (31.5)	
I like learning English			.006
Yes	69 (75.8)	22 (24.2)	
No	4 (36.4)	7 (63.6)	
English is useful for the medical field			>.99
Yes	70 (71.4)	28 (28.5)	
No	3 (75.0)	1 (25)	
English should be the language of higher education			.02
Yes	68 (75.6)	22 (24.4)	
No	7 (58.3)	5 (41.7)	

a Not applicable

For the variable “English is useful for the medical field,” 96.1% (98/102) of respondents agreed with the statement, and 71.4% (70/98) of those who agreed chose EMI. The *P* value for this variable did not reach statistical significance, a mathematical outcome attributed to the strong homogeneity of responses and subsequent reduced variance. Given the resulting low expected cell counts, the Fisher exact test was used. Calculation of the Cramer *V* coefficient revealed a negligible mathematical association (Cramer *V*=0.015).

To further evaluate predictors of EMI choice, univariate and multivariate logistic regression analyses were performed (Table 7). The univariate regression identified 3 statistically significant predictors: course year (fourth vs third; OR 23.14, 95% CI 2.85‐187.64; *P*=.003), “I like learning English” (OR 5.49, 95% CI 1.47‐20.52; *P*=.01), and “English should be the language of higher education” (OR 4.33, 95% CI 1.25-15.02; *P*=.02).

In the final multivariate model (following backward stepwise selection and the forced entry of sex and “English is useful for the medical field”), only 2 variables maintained a statistically significant positive influence on EMI choice after adjustment: course year (fourth vs third; aOR 22.12, 95% CI 2.56‐190.84; *P*=.005) and “I like learning English” (aOR 5.12, 95% CI 1.05‐25.03; *P*=.04). The 2 variables included based on theoretical relevance did not reach statistical significance in the final model.

**Table 7.** Associated factors with English-medium instruction (EMI) choice: univariate and multivariate logistic regression models.

Associated factors	Univariate model			Multivariate model		
	OR^a^ (95% CI)		*P* value	aOR^b^ (95% CI)		*P* value
Sex	0.58 (0.23-1.48)		.25	0.33 (0.10-1.10)		.07
Course year						
Fourth vs third	23.14 (2.85-187.64)		.003	22.12 (2.56-190.84)		.005
Fifth vs third	2.00 (0.64-6.28)		.24	1.89 (0.52-6.88)		.33
Sixth vs third	2.36 (0.64-8.70)		.20	2.22 (0.53-9.28)		.28
English certification	1.76 (0.63-4.92)		.28	1.75 (0.52-5.90)		.37
I like learning English	5.49 (1.47-20.52)		.01	5.12 (1.05-25.03)		.04
English is useful for the medical field	0.83 (0.08-8.36)		.88	0.46 (0.04-5.39)		.54
English should be the language of higher education	4.33 (1.25-15.02)		.02	2.29 (0.48-10.88)		.30

a OR: odds ratio.

b aOR: adjusted odds ratio.

## Discussion

### Students’ Attitudes Toward EMI

This study revealed generally positive attitudes toward EMI among medical students at FMPR. Participants recognized the profound utility of English for the medical field, particularly its role in facilitating access to medical knowledge, expanding professional opportunities, and fostering international mobility. The advantage-related dimensions identified in this study are consistent with previous research reporting perceived benefits in English proficiency, access to up-to-date medical literature, and enhanced career prospects [2,21,23,32]. Conversely, the disadvantage-related dimensions, especially difficulties in understanding disciplinary content in English and the increased cognitive burden associated with learning in a new instructional medium, align with challenges previously documented in EMI contexts [2,7]. Overall, strong support for EMI within a socioeconomically diverse public institution such as FMPR challenges the view that EMI is confined to private elites and suggests that implementing EMI in public medical schools may broaden access to global medical education.

The respondents’ favorable perspective is strongly driven by the academic and professional necessities of the medical field. Because international medical communication predominantly occurs in English, EMI enables students to access the latest clinical studies, stay updated with evidence-based practices, and collaborate with the global medical community, which is pivotal for professional growth [7,24]. This study demonstrated a significant developmental trend: as students advanced in their training, their preference for an EMI curriculum increased substantially. This reflects the pressing need to access global medical literature for graduation theses and specialization, as the vast majority of indexed medical research is currently published in English [1]. Moreover, students’ interest in learning English was a strong predictor of EMI choice. As Nguyen [24] pointed out, EMI facilitates the acquisition of English medical terminology indispensable for international scientific exchange. Similar favorable attitudes toward EMI, despite persistent language and cognitive challenges, have been reported among medical students in Saudi Arabia and in Moroccan health sciences programs, where students recognized EMI’s advantages for accessing international literature and employability while expressing concerns about comprehension difficulties and the need for pedagogical support [8,26,32].

### Implications for Policy and Practice

A critical insight from this study is the capacity of EMI programs to contribute to educational democratization within public medical faculties. Historically, linguistic background has played a key role in academic performance [27]. Under the traditional French-medium model, graduates of Arabic-medium public schools often faced significant language barriers, whereas students from elite French-medium private schools enjoyed a systemic advantage [9,37]. By contrast, English occupies a more neutral linguistic position [33]. Through digital media and informal exposure, many students, regardless of their prior schooling, develop similar basic interpersonal communication skills in English, thereby reducing initial language-related disparities [13]. In our cohort, the vast majority of students recognized the instrumental value of EMI, particularly its role in securing better professional prospects and facilitating international mobility [16,32]. Consequently, integrating EMI in socioeconomically diverse public institutions like FMPR may help mitigate entrenched disparities. Unlike private institutions where EMI often serves the privileged, public EMI programs have the potential to democratize access to global medical opportunities, including compliance with WFME- and Educational Commission for Foreign Medical Graduates–related standards [9,42,43].

This potential for democratization aligns with recent macroeducational policies. Following Brexit, Morocco and the United Kingdom signed educational agreements aiming to enhance international research collaboration and English integration [39]. Capitalizing on these frameworks, several Moroccan private universities introduced EMI medical programs [32,33]. To prevent widening the public-private educational divide, the 2023 United Kingdom-Morocco Memorandum of Understanding targeted English integration within public universities [40]. While implementing EMI in resource-constrained public sectors poses significant structural challenges that mirror global trends, our findings at FMPR indicate a remarkable student receptiveness toward EMI despite existing sectoral barriers [4].

### Comparison With Prior Work

In the context of global trends, Moroccan students’ positive attitudes align with those of their regional peers in the Middle East and North Africa region, such as Algeria and Libya, and broader African contexts [22,29-31]. Interestingly, this enthusiasm is comparable to trends observed in high-income countries like Japan [3]. More broadly, it aligns with an ongoing reorientation of language-in-education policies across North Africa, where English is increasingly viewed as a strategic alternative to French [11]. Despite a shared legacy that established French as the dominant instructional language, there is a growing tendency toward the adoption of English [37]. Unlike French, which carries colonial connotations and functions as a marker of elite status, English is widely perceived in Morocco as a neutral gateway to international mobility [32]. The receptiveness to EMI is strongly supported by a young population highly favorable to English [14]. As documented by Belhiah et al [34] in a Moroccan multicenter study, students expressed a passion for English as a subject worth studying.

Cultural concerns regarding the loss of native language were notably minimal. This *p*attern reflects an additive approach to multilingualism where English is embraced primarily for its instrumental value in facilitating global medical integration, as the French medium of higher education is already a nonnative language without any detrimental impact on Moroccan students’ cultural identity. However, while digital media facilitates the robust development of basic interpersonal communication skills, this conversational fluency does not translate to the Cognitive Academic Language Proficiency (CALP) required for medical EMI [12,13]. At the institutional level, practical concerns persist regarding the dual cognitive load of simultaneously mastering complex medical content and improving English proficiency. As highlighted by the Sweller cognitive load theory [48], this dual processing can overburden working memory, underscoring the urgent need for structured pedagogical support within EMI programs. In this sense, EMI in a public Francophone medical school appears to have a conditional democratizing potential: it may widen access to global medical education for students from diverse backgrounds, while simultaneously requiring careful management of language demands and cognitive load.

### Limitations

This study has limitations that should be acknowledged. First, because existing instruments do not capture the unique sociolinguistic reality of Moroccan public medical schools (Arabic L1, French as the medium of instruction, and English emerging), our questionnaire remains exploratory. While the perceived proficiency scale (α=0.949) demonstrated excellent reliability, the attitudinal dimensions showed moderate internal consistency (α=0.670); although acceptable for exploratory research and novel scale development [46,47], this warrants careful interpretation. Second, self-reported data may not fully reflect students’ actual English proficiency or academic performance under EMI conditions. Third, the cross-sectional design precludes longitudinal analysis. Finally, while the monocentric sample provides detailed insights into FMPR, it limits generalizability to other medical schools or disciplines.

Despite these limitations, this study elucidates critical EMI trends within a Francophone public medical faculty, contributing context-specific evidence to inform medical education policy in comparable global settings.

### Conclusions

Integrating EMI into public medical faculties in Morocco is perceived more as an opportunity than as a constraint. Most second-cycle FMPR students expressed positive attitudes toward EMI and generally favorable self-perceptions of their English proficiency. Support for English was stronger among advanced students, driven by a heightened awareness of its role in international professional development. Framed as a pragmatic global lingua franca rather than a colonial legacy, English enjoys broad acceptance across diverse social backgrounds. Within this context, EMI holds significant potential to support a more equitable distribution of educational and professional opportunities.

These findings offer significant implications for theory, policy, and educational practice. Theoretically, this study reinforces a constructivist perspective in which students from the Global South actively embrace EMI as a tool for epistemic justice rather than passively receiving it as a linguistic imposition. At the policy level, the strong student receptiveness provides an evidence-based mandate for decision-makers to formalize EMI integration in public medical education. Aligning with Morocco’s 2022‐2026 Educational Roadmap, adopting EMI in public faculties is crucial to prevent a two-tier health care education system, ensuring that public school graduates are not excluded from global mobility and international accreditations compared to their privately educated peers.

Practically, to successfully implement medical EMI in the Moroccan context, institutions must address the observed discrepancy between high enthusiasm and weaker self-reported academic English proficiency in a subset of students. Implementation strategies should include English for Medical Purposes preparatory courses, scaffolded bilingual instruction, continuous pedagogical training for medical educators, and targeted English language support for students to mitigate their cognitive burden.

Future research must adopt a phased approach to EMI implementation. The preimplementation phase should inform evidence-based curriculum design. Mixed-methods and qualitative investigations are essential to explore students’ underlying concerns, clarify factors moderating their EMI preferences, and identify unanticipated pedagogical barriers prior to implementation. The authors are currently addressing these imperatives through a comprehensive needs analysis of both students and medical educators to guide targeted institutional interventions. Subsequently, postimplementation longitudinal research will be critical to evaluate students’ lived experiences and assess EMI’s impact on academic performance and cognitive load. Ultimately, provided its integration is gradual, adequately resourced, and responsive to local constraints, EMI offers a credible pathway to equity and global competitiveness in Moroccan public medical education.

## Supplementary material

10.2196/95392 Multimedia Appendix 1 Questionnaire.

10.2196/95392 Checklist 1 STROBE (Strengthening the Reporting of Observational Studies in Epidemiology) Checklist.

## Abbreviations

aOR: adjusted odds ratio
CEFR: Common European Framework of Reference for Languages
EFA: exploratory factor analysis
EMI: English-medium instruction
FMPR: Faculty of Medicine and Pharmacy of Rabat
KMO: Kaiser-Meyer-Olkin
OR: odds ratio
STROBE: Strengthening the Reporting of Observational Studies in Epidemiology
WFME: World Federation for Medical Education
