# Supplementary material for: Medical Students’ Perceptions and Attitudes Toward English as a Medium of Instruction at the Faculty of Medicine and Pharmacy of Rabat: Cross-Sectional Study
Source: JMIR Form Res. 2026 Jul 23;10:e95392. doi: 10.2196/95392 (PMC13394863; doi:10.2196/95392)
Supplement: Multimedia Appendix 1 [file formative-v10-e95392-s001.docx]

**Questionnaire**

Dear participant,

This questionnaire is part of a research project on the use of the English language (EL) as a means of instruction in your faculty (EMI: English as a medium of instruction). Some questions are in the form of Likert scales while others have multiple choices, allowing you to check the answers that seem most appropriate to you. Your contribution is invaluable to the completion of this research project and is highly appreciated. Your anonymous answers will be held in strict confidentiality and will be used only for the purposes of this study. The results will be reported in aggregate form only, and cannot be identified individually. Thank you for your collaboration.

The researcher

**I. DEMOGRAPHIC DATA**

**1. Age**

..... Years

**2. Gender**

□ Male □ Female

**3. Course year**

□ 3^rd^ year □ 4^th^ year □ 5^th^ year □ 6^th^ year □ 7^th^ year

**II. PERCEIVED PROFICIENCY IN ENGLISH LANGUAGE**

**4. Do you speak EL?**

□ Yes □ No

**5. At what grade level did you start learning EL?**

.........

**6. Have you got a certified EL level?**

□ Yes □ No

**7. If you checked Yes above, please check your certified level below.**

□ A1 □ A2 □ B1 □ B2 □ C1 □ C2

**8. General EL**

***Instructions:* On a scale from 0 to 5, select your level of proficiency in EL in the following areas:**

**0…………………1……….……….2……….…….3………………….4………..…….….5**

**None Basic Average Quite Good Good Excellent**

| Speaking in regular everyday interactions | 0……..1……..2……..3……..4……..5 |
| --- | --- |
| Listening in regular daily interactions | 0……..1……..2……..3……..4……..5 |
| Reading about general topics of interest to me (Newspapers, magazines, books) | 0……..1……..2……..3……..4……..5 |
| Communicate in writing on social media platforms | 0……..1……..2……..3……..4……..5 |

**9. Academic EL**

***Instructions:* On a scale from 0 to 5, select your level of proficiency in EL for academic studies in the field of medicine.**

**0…………………1……….……….2……….…….3 …………..……. 4………..……….5**

**None Basic Average Quite Good Good Excellent**

| Discuss technical ideas in the field of medicine. | 0……..1……..2……..3……..4……..5 |
| --- | --- |
| Prepare and deliver a presentation about a medical issue. | 0……..1……..2……..3……..4……..5 |
| Understand videos about medical issues | 0……..1……..2……..3……..4……..5 |
| Read medicine textbooks in EL | 0……..1……..2……..3……..4……..5 |
| Write class assignments in EL about topics in medicine | 0……..1……..2……..3……..4……..5 |
| Communicating in EL with my course instructors | 0……..1……..2……..3……..4……..5 |
| Write a scientific article for publication in a medical journal | 0……..1……..2……..3……..4……..5 |

**10. Exposure to EL**

***Instructions:* Please, rate to what extent you are exposed to EL in the following contexts.**

| Interacting with friends | Never…rarely…sometimes…often…always |
| --- | --- |
| Watching movies | Never…rarely…sometimes…often…always |
| Listening to the radio/music | Never…rarely…sometimes…often…always |
| Listening to podcasts | Never…rarely…sometimes…often…always |
| Reading | Never…rarely…sometimes…often…always |
| Free writing | Never…rarely…sometimes…often…always |
| Private English classes | Never…rarely…sometimes…often…always |

**11. What strategies do you use to improve your EL skills?**

□ Private language classes

□ Reading

□ Discussion

□ Watching Movies

□ Listening to music

□ Using English online

□ Others (Please specify) ………………………………………….

**III. LANGUAGE PERCEPTIONS AND ATTITUDES**

***12. Instructions: Please use the scale below to rate the extent to which you agree/disagree with the following:***

**1……….………..….2……………..….3 ………..….....…. 4……..…..……….5**

**Strongly Disagree Disagree Neutral Agree Strongly Agree**

| I like learning EL | 1………....2……..…..3….….…..4….……..5 |
| --- | --- |
| I like to use EL | 1………....2……..…..3….….…..4….……..5 |
| EL is very useful for work in Morocco | 1………....2……..…..3….….…..4….……..5 |
| EL is useful for medical scientific field | 1………....2……..…..3….….…..4….……..5 |
| EL should be the language of higher education | 1………....2……..…..3….….…..4….……..5 |
| We should have more courses using EMI in higher education | 1………....2……..…..3….….…..4….……..5 |
| If an EL program is offered in my faculty, I will definitely go for it. | 1………....2……..…..3….….…..4….……..5 |
| Multilingualism offers me advantages | 1………....2……..…..3….….…..4….……..5 |
| We should keep both French language and EL as mediums of instruction in higher education | 1………....2……..…..3….….…..4….……..5 |

**IV. NEEDS OF EMI**

***13. Instructions: Please use the scale below to rate the extent to which you agree/disagree with the following:***

**1……….………..….2……………..….3 ………..….....…. 4……..…..……….5**

**Strongly Disagree Disagree Neutral Agree Strongly Agree**

**For medical studies using EMI, I need training in the following skills and areas:**

| Academic Speaking (e.g. Presentations) | 1………....2……..…..3….….…..4….……..5 |
| --- | --- |
| Academic Listening (e.g. Lectures) | 1………....2……..…..3….….…..4….……..5 |
| Academic Reading (e.g. Textbooks) | 1………....2……..…..3….….…..4….……..5 |
| Academic Writing (e.g. Assignments) | 1………....2……..…..3….….…..4….……..5 |
| Academic Vocabulary | 1………....2……..…..3….….…..4….……..5 |
| Grammar | 1………....2……..…..3….….…..4….……..5 |

**14. What resources can support your medical studies using EMI?**

***Instructions: Please use the scale below to rate the extent to which you agree/disagree with the following:***

**1……….………..….2……………..….3 ………..….....…. 4……..…..……….5**

**Strongly Disagree Disagree Neutral Agree Strongly Agree**

| Access to medical EL online courses |  |
| --- | --- |
| Access to sites for medical students | 1………....2……..…..3….….…..4….……..5 |
| Access to medical EL tutorials, with activities and exercises for doctors | 1………....2……..…..3….….…..4….……..5 |
| Sites for general EL |  |
| Sites for online grammar with exercises | 1………....2……..…..3….….…..4….……..5 |
| Subscriptions in international professional associations | 1………....2……..…..3….….…..4….……..5 |

**15. What difficulties do you think you may encounter if EL is adopted as a medium of instruction at your faculty?**  **(Please check all that apply).**

□ Understanding lectures

□ Understanding EL medical textbooks

□ Participating in class discussions

□ Writing assignments in EL

□ Preparing and delivering presentations in EL

□ Others (Please specify)

**16. Have you received any training in studying using EMI? If yes, what type of training? (Please check all that apply)**

□ No, I haven’t received any training

□ General EL courses

□ Academic EL courses

□ E-learning platform courses

□ Video-conferencing courses

□ Others (Please specify): …………………………………………..

**17. Do you think that EMI could help you improve your EL skills?**

□ Strongly disagree □ Disagree □ Neutral □ Agree □ Strongly agree

**18. How important is EMI to your career prospects?**

□ Unimportant □ Slightly Important □ Moderately Important □ Important □ Very Important

**19. What do you see as the benefits of EMI?**

□ To improve my level of EL skills (listening, speaking, oral interactions, presentations, debates, reading, writing)

□ To improve my learning of the medical content subjects

□ To have better professional opportunities

□ To have more opportunities regarding international mobility (study or work abroad)

□ Others (Please, specify) ……………………………………………….

**20. In your opinion, what are the disadvantages EMI? (Please check all that apply)**

□ Loss of mother tongue

□ Loss of local culture

□ Loss of identity

□ The content subjects are more difficult to learn in English

□ Other (Please specify): ………………………………………………

**21. What do you think about the use of EMI in higher education in general?**

□ Strongly disagree □ Disagree □ Neutral □ Agree □ Strongly agree

**22. If I have the choice, I will opt for an EMI program if**

**Instructions: Please use the scale below to rate the extent to which you agree/disagree with the following statements:**

**1……….………..….2……………..….3 ………..….....…. 4……..…..……….5**

**Strongly Disagree Disagree Neutral Agree Strongly Agree**

| It is exclusively in EL | 1………....2……..…..3….….…..4….……..5 |
| --- | --- |
| It offers a bilingual FL-EL training | 1………....2……..…..3….….…..4….……..5 |
| It offers regular classes in academic EL every year | 1………....2……..…..3….….…..4….……..5 |
| It offers regular classes in medical EL every year | 1………....2……..…..3….….…..4….……..5 |
| If there is a certified EL proficiency requirement for incoming students (IELTS, TOEFL). | 1………....2……..…..3….….…..4….……..5 |
| The lectures are in EL | 1………....2……..…..3….….…..4….……..5 |
| The examinations are in EL | 1………....2……..…..3….….…..4….……..5 |
| If translation of concepts and use of FL is tolerated in class | 1………....2……..…..3….….…..4….……..5 |
| Lectures discussions are exclusively in EL | 1………....2……..…..3….….…..4….……..5 |

**23. Do you have any other suggestions or comments regarding EMI?**

**………………………………………………………………………………………………………………………………………………………………………………………………………………**
