# Supplementary material for: Medical Students’ Perceptions and Attitudes Toward English as a Medium of Instruction at the Faculty of Medicine and Pharmacy of Rabat: Cross-Sectional Study
Source: JMIR Form Res. 2026 Jul 23;10:e95392. doi: 10.2196/95392 (PMC13394863; doi:10.2196/95392)
Supplement: Checklist 1 [file formative-v10-e95392-s002.doc]

**STROBE Checklist M 95392 JMIR Formative Research**

|  | Item No | Recommendation |
| --- | --- | --- |
| **Title and abstract** | 1 | (*a*) Indicate the study’s design with a commonly used term in the title or the abstract. Reported on pages: Title p.1; Abstract p.1-2. |
|  |  | (*b*) Provide in the abstract an informative and balanced summary of what was done and what was found  Reported on page p.1-2. |
| Introduction | | |
| Background/rationale | 2 | Explain the scientific background and rationale for the investigation being reported.  Reported on pages: p.2-3. |
| Objectives | 3 | State specific objectives, including any prespecified hypotheses.  Reported on page: p.3. |
| Methods | | |
| Study design | 4 | Present key elements of study design early in the paper  Reported on page: p.3. |
| Setting | 5 | Describe the setting, locations, and relevant dates, including periods of recruitment, exposure, follow-up, and data collection  Reported on pages: p.3-4. |
| Participants | 6 | (*a*) Give the eligibility criteria, and the sources and methods of selection of participants  Reported on page: p.3. |
| Variables | 7 | Clearly define all outcomes, exposures, predictors, potential confounders, and effect modifiers. Give diagnostic criteria, if applicable  Reported on page: p.4. |
| Data sources/ measurement | 8* | For each variable of interest, give sources of data and details of methods of assessment (measurement). Describe comparability of assessment methods if there is more than one group  Reported on page: p.4. |
| Bias | 9 | Describe any efforts to address potential sources of bias  Reported on pages: p.4-9 |
| Study size | 10 | Explain how the study size was arrived at  Reported on page: p.3. |
| Quantitative variables | 11 | Explain how quantitative variables were handled in the analyses. If applicable, describe which groupings were chosen and why  Reported on page: p.4. |
| Statistical methods | 12 | (*a*) Describe all statistical methods, including those used to control for confounding  Reported on page: p.4. |
|  |  | (*b*) Describe any methods used to examine subgroups and interactions  Reported on page: p.4. |
|  |  | (*c*) Explain how missing data were addressed  Reported on page: p.4. |
|  |  | (*d*) If applicable, describe analytical methods taking account of sampling strategy  Reported on page: p.3. |
|  |  | (*e*) Describe any sensitivity analyses  Reported on page: NA |
| Results | | |
| Participants | 13* | Report numbers of individuals at each stage of study—eg numbers potentially eligible, examined for eligibility, confirmed eligible, included in the study, completing follow-up, and analysed  Reported on page: p.4. |
|  |  | Give reasons for non-participation at each stage  Reported on page: p.4. |
|  |  | (c) Consider use of a flow diagram  Reported on page: NA |
| Descriptive data | 14* | Give characteristics of study participants (eg demographic, clinical, social) and information on exposures and potential confounders  Reported on page: p.5. |
|  |  | (b) Indicate number of participants with missing data for each variable of interest  Reported on page: p.5. |
| Outcome data | 15* | Report numbers of outcome events or summary measures  Reported on page: p.6. |
| Main results | 16 | Give unadjusted estimates and, if applicable, confounder-adjusted estimates and their precision (eg, 95% confidence interval). Make clear which confounders were adjusted for and why they were included  Reported on pages: p.7-8. |
|  |  | (*b*) Report category boundaries when continuous variables were categorized  Reported on page: p.4. |
|  |  | (*c*) If relevant, consider translating estimates of relative risk into absolute risk for a meaningful time period  Reported on page: NA. |
| Other analyses | 17 | Report other analyses done—eg analyses of subgroups and interactions, and sensitivity analyses  Reported on page: NA. |
| Discussion | | |
| Key results | 18 | Summarise key results with reference to study objectives  Reported on pages: p.8-9. |
| Limitations | 19 | Discuss limitations of the study, taking into account sources of potential bias or imprecision. Discuss both direction and magnitude of any potential bias  Reported on page: p.9. |
| Interpretation | 20 | Give a cautious overall interpretation of results considering objectives, limitations, multiplicity of analyses, results from similar studies, and other relevant evidence  Reported on pages: p.8-9-10 |
| Generalisability | 21 | Discuss the generalisability (external validity) of the study results  Reported on page: p.10. |
| Other information | | |
| Funding | 22 | Give the source of funding and the role of the funders for the present study and, if applicable, for the original study on which the present article is based  Reported on page: NA. |

*Give information separately for exposed and unexposed groups.

**Note:** An Explanation and Elaboration article discusses each checklist item and gives methodological background and published examples of transparent reporting. The STROBE checklist is best used in conjunction with this article (freely available on the Web sites of PLoS Medicine at http://www.plosmedicine.org/, Annals of Internal Medicine at http://www.annals.org/, and Epidemiology at http://www.epidem.com/). Information on the STROBE Initiative is available at www.strobe-statement.org.
